# Impact of Biofloc on Life Characteristics, Fecundity, and Innate Immunity of Fairy Shrimp (*Branchinella thailandensis*, Sanoamuang, Saengphan and Murugan, 2002)

**DOI:** 10.3390/biology15080650

**Published:** 2026-04-20

**Authors:** Watcharapong Tharapan, Pattira Kasamesiri, Zhongliang Wang, Laorsri Sanoamuang, Wipavee Thaimuangphol

**Affiliations:** 1 Applied Animal and Aquatic Sciences Research Unit, Division of Fisheries, Faculty of Technology, Mahasarakham University, Maha Sarakham 44150, Thailand; 63010854002@msu.ac.th; 2 Faculty of Technology, Mahasarakham University, Maha Sarakham 44150, Thailand; pattira@msu.ac.th; 3 College of Fisheries, Guangdong Ocean University, Zhanjiang 524088, China; zhongliangwang@vip.163.com; 4 Guangdong Provincial Key Laboratory of Aquatic Animal Disease Control and Healthy Culture, Zhanjiang 524088, China; 5 International College, Khon Kaen University, Khon Kaen 40002, Thailand; la_orsri@kku.ac.th; 6 Applied Taxonomic Research Center, Department of Biology, Faculty of Science, Khon Kaen University, Khon Kaen 40002, Thailand

**Keywords:** Anostraca, carotenoid, antioxidant activity, fisheries, live feed

## Abstract

This study examined the life characteristics, fecundity, and innate immune response of fairy shrimp (*Branchinella thailandensis* Sanoamuang, Saengphan and Murugan, 2002) cultivated on *Chlorella vulgaris*, biofloc, dried powder *Spirulina* sp., and a formulated feed. These feeds were selected to compare traditional live feed (*Chlorella vulgaris*), microbial-based feed (biofloc), and alternative dry feed in order to identify a sustainable feeding strategy for fairy shrimp culture. The lifespans of male and female fairy shrimp reared on the biofloc diet were the longest at 44 ± 6.00 and 44.33 ± 4.67 days, respectively. Fairy shrimp reared on the biofloc diet demonstrated significantly enhanced growth, with lengths of 25.66 ± 1.75 mm in males and 27.60 ± 2.08 mm in females. The highest fecundity was also observed in the biofloc treatment, with 25 ± 6.65 broods per female, an average of 229.57 ± 2.08 eggs per brood, and a total of 5726.33 ± 1518.11 eggs per female over their lifespan. Fairy shrimp fed with *Chlorella vulgaris*, biofloc, and *Spirulina* sp. showed significantly high levels of superoxide dismutase (SOD) and lysozyme activities compared to those fed on a formulated feed, while malondialdehyde (MDA) levels, a marker of oxidative stress, were lower in these three groups. Results suggested that biofloc was a viable feed option for *B. thailandensis*, which positively influenced growth, lifespan, and fecundity while enhancing the innate immune function.

## Simple Summary

The different feed types significantly influence the biological performance of the Thai fairy shrimp, *Branchinella thailandensis*. Fairy shrimp fed biofloc exhibited prolonged lifespan, larger body size, and high survival rates comparable to those of the control group fed *Chlorella*. The biofloc-fed group showed markedly higher fecundity compared with the other treatments. Fairy shrimp fed *Chlorella*, biofloc, and *Spirulina* also showed similar levels of total carotenoids, lysozyme activity, and antioxidant enzymes including superoxide dismutase (SOD) and catalase (CAT), as well as comparable levels of oxidative stress indicated by malondialdehyde (MDA). In contrast, fairy shrimp fed commercial powdered feed showed the lowest survival rate, reduced fecundity, and the lowest levels of immune and antioxidant enzyme activities, while exhibiting the highest oxidative stress levels.

## 1. Introduction

Aquaculture expansion has matched the increasing demand for production inputs, including high-quality aquatic offspring, effective farm management systems, and nutritionally efficacious feed. Live food for aquatic animals is particularly important, and its high nutritional value significantly influences growth and survival rates.

Live food plays an important role in aquaculture due to its high nutritional value [1] and its beneficial effects on aquatic animals. It provides essential nutrients and stimulates the immune system [2], enhances appetite [3], and improves the reproductive system) [4]. Common live foods used in aquaculture include bloodworm, rotifer, water flea and *Artemia*. Among these, *Artemia* is the most widely used due to its rich nutritional profile [1,5]. However, the importation of *Artemia* presents a significant challenge in Thailand, incurring substantial costs [6], which limit the sustainability of aquaculture practices in the region. Therefore, there is a pressing need to explore alternative live food sources to replace *Artemia*. One promising alternative is the fairy shrimp, which belongs in the same group as *Artemia*.

Fairy shrimp are native to Thailand and inhabit temporary freshwater ecosystems, making them a cost-effective and locally available alternative. Utilizing fairy shrimp as live feed reduces the dependence on imported *Artemia* and improves sustainability in local aquaculture systems [6]. Fairy shrimp resemble small freshwater crustaceans. They are characterized by their shell-less, translucent bodies and are found throughout Thailand. They have high levels of protein and carotenoids and are valued for their nutritional content. Three species have been identified in Thailand as *Streptocephalus sirindhornae*, *Branchinella thailandensis*, and *Streptocephalus siamensis* [7]. Of these, *S. sirindhornae* and *B. thailandensis* are the most widely cultivated. *B. thailandensis* demonstrates rapid growth, attains a larger body size [8], and has elevated carotenoid levels. Fairy shrimp contain high amounts of carotenoids, including beta-carotene, astaxanthin, lutein, and canthaxanthin. Beta-carotene and astaxanthin are the most important nutrients and fairy shrimp have all the essential amino acids that animals need to grow [9,10]. Consequently, they are used as both live and frozen ornamental fish feed and have been shown to enhance growth performance and coloration [11]. The nutritional value of fairy shrimp has made them increasingly attractive in domestic and international aquaculture markets

Despite this potential, fairy shrimp cultivation faces challenges, particularly in feeding practices. Currently, the primary feed used is green algae, especially *Chlorella vulgaris* [12], due to its small cell size, single cell, high nutritional value, and rich amino acid profile, including threonine, lysine, and leucine [13,14]. However, *Chlorella* cultivation is impacted by inadequate light, cold seasonal temperatures, high stocking densities, and the accumulation of ammonia, which can be toxic to fairy shrimp. As a result, alternative feed sources have been explored, including photosynthetic bacteria [15], yeast, fermented microbial water [9], and wastewater from fishponds [16], but these alternatives often lack the nutritional adequacy of *Chlorella vulgaris*.

In their natural environment, fairy shrimp consume a diverse range of food, including phytoplankton and zooplankton [17]. Hence, in the cultivation of fairy shrimp, diversifying the diet is essential. Utilizing a range of bacteria with distinct groups and benefits promotes better growth outcomes compared to a single-feed approach, as these diverse bacteria enhance nutrient availability and improve the overall health of the fairy shrimp. The concept of employing diverse bacterial groups originates from biofloc technology, a widely adopted practice in fish and shrimp farming. The application of biofloc technology in fairy shrimp cultivation offers an alternative feed source with nutritional value comparable to or exceeding that of *Chlorella* algae.

Biofloc is a modern technology involving the dense growth of beneficial microbial communities that enhance aquaculture productivity. Biofloc systems typically include protozoa, microalgae, fungi, and predominantly heterotrophic bacteria suspended in the water column. These microorganisms break down organic waste and significantly reduce the accumulation of ammonia, a metabolic byproduct harmful to aquatic organisms [18,19]. Biofloc also serves as an alternative protein source [20,21]. Inducing biofloc requires adding organic carbon and maintaining consistent aeration to stimulate microbial proliferation [22]. The benefits of biofloc systems include improved immune responses, accelerated growth [23], ammonia conversion to protein-rich biomass, and reduced water exchange [24]. Many studies have demonstrated that biofloc technology can improve water quality, help aquatic animals grow, and strengthen the immune systems of different species, such as white shrimp [25], tilapia [26,27], and giant freshwater prawn [28].

Despite these benefits, the application of biofloc in zooplankton, particularly fairy shrimp, remains underexplored. The diverse living organisms in biofloc systems serve as a natural food source, and integrating biofloc technology into fairy shrimp cultivation could enhance water quality, improve nutritional intake and promote immune function. Therefore, this study evaluated the feasibility of using biofloc technology as a novel feeding approach in the cultivation of fairy shrimp to develop a sustainable, nutritionally balanced, and cost-effective live feed source to support the aquaculture industry.

## 2. Materials and Methods

### 2.1. Ethical Approval for Animal Use

This research was conducted in strict accordance with the recommendations for the use of animals regulated by the Institute of Animals for Scientific Purposes Development (IAD) of Thailand. The Ethics Committee at Mahasarakham University (IACUC-MSU-023-003/2025 and 28 March 2025) approved the fish handling and experimental protocols.

### 2.2. Animal Preparation

Eggs of fairy shrimp were obtained from the Applied Taxonomic Research Center, Department of Biology, Faculty of Science, Khon Kaen University, Khon Kaen, Thailand. Nauplii were hatched using dechlorinated tap water and fed 1 × 10^6^ cells mL^−1^ with *Chlorella vulgaris* twice a day, following the method described by Dararat et al. [8] until the animals reached 5 days old.

### 2.3. Preparation Experimental Diets

Four fairy shrimp diets were applied as *Chlorella vulgaris*, biofloc, dried *Spirulina* sp., and a dried commercial diet. *Chlorella vulgaris* were cultured following the method of Dararat et al. [8]. Before feeding, *Chlorella vulgaris* containing 40.82% protein and 0.85% lipid were centrifuged (Z206A, Hermle Labortechnik, Wehingen, Germany) at 3000 rpm. The fairy shrimp were fed 1 × 10^6^ cells mL^−1^ twice per day [8]. Biofloc containing 29.04% protein and 0.71% lipid was prepared using 200 juveniles of *Oreochromis niloticus* with an average weight of 7.53 ± 2.52 g and fed with a 32% crude protein commercial diet (Betagro, Bangkok, Thailand) at a ratio of 3% of the biomass, serving as a nitrogen source for biofloc development. The stability of the biofloc level in biofloc ponds was maintained by the daily addition of molasses as a carbon source, using a C:N ratio of 15:1. Following the method of Avnimelech [29], the total ammonia nitrogen concentration (TAN) was 0 mg L^−1^ and total suspended solids (TSS) ranged between 250 and 500 mg L^−1^ throughout the experiment [30]. The biofloc was passed through a 60-micron filter before feeding to the fairy shrimp.

Dried powder *Spirulina* containing 40.97% protein and 0.54% lipid was purchased from Wellgate Distribution Co., Ltd., Bangkok, Thailand, while commercial diet powder containing 24.80% protein and 8.10% lipid as the formulated feed was purchased from Thai Union Feedmill PCL, Samut Sakhon, Thailand. Both the dried diets were fed to the fairy shrimp at 3.0 mg dry weight individual^−1^ following Chaoruangrit et al. [31], after passing through a 60-micron filter.

### 2.4. Experimental Design

The experiment setup was divided into four feeding groups with three replicates. Male and female fairy shrimp pairs were cultured in 500 mL plastic bowls containing 350 mL of tap water. Water in the experimental units was removed daily and replaced with freshly prepared water, and the life history characteristics of the fairy shrimps as lifespan, body length, maturation, and fecundity were recorded daily.

For the second experiment on mass culture of fairy shrimp, each treatment was conducted in triplicate (three independent tanks per treatment). In each replicate, 500 individuals were randomly selected and reared in a 100 L plastic tank containing 50 L of tap water. After 15 days, the fairy shrimp were collected for nutritional, proximate, and carotenoid analyses. For proximate composition, carotenoid, and immune analyses, sample was taken from each replicate tank. Three samples (*n* = 3) per treatment were analyzed, which explains the statistical representation.

### 2.5. Biological Characteristics Study

When eggs were first presented in the brood pouches of the females and full-grown antennae were formed on the males, male-female pairs of fairy shrimp were cultured in 500 mL plastic containers, with observations continued until the animals died. The growth, lifespan, and fecundity of the fairy shrimp were observed daily. Fairy shrimp body length was measured every six days from the tip of the head to the posterior margin of the caudal furcae using a vernier caliper, with the survival rate observed daily.

The growth parameters of the fairy shrimp in each tank were measured and reported at 6-day intervals. Body length (mm) and survival rate (SR) were determined using the following equations [32]:

Body length (mm) = Measure the tip of the head to the posterior end of the telson

Survival rate (%) = (final shrimp number/initial shrimp number) × 100

All the deposited eggs from each female were collected and counted under a stereo microscope to determine egg production.

### 2.6. Proximate Analysis and Carotenoid Content

The crude protein, crude lipid, and ash of the fairy shrimp were determined following the method of the Association of Official Analytical Chemists [33]. Crude protein (N × 6.25) was determined by the Kjeldahl method after acid digestion using a Kjeldahl system. Crude lipid was determined by the hexane extraction method using a Soxhlet extractor. Ash content was determined by heating in a muffle furnace (MF-2011, Daeyang, Gyeonggi, Republic of Korea) at 600 °C for 2 h.

The carotenoid concentrations in the adult fairy shrimp were determined by homogenization and suspension in acetone, following the methods described by Rodriguez-Amaya and Kimura [34]. Briefly, 0.2–0.5 g of fairy shrimp samples was homogenized and repeatedly extracted in acetone until no color was detected. The acetone extracts were then collected and evaporated under vacuum using a rotary evaporator (RC900; KNF Neuberger GmbH, Freiburg im Breisgau, Germany). The dried extract was added with petroleum ether and saponified with KOH. The alkali in the extract was eliminated by washing with distilled water. The carotenoid concentrate was measured in petroleum ether and made up to a known final volume, using a GENESYS™ 20 visible spectrophotometer (Thermo Fisher Scientific Inc., Waltham, MA, USA) at 450 nm.

### 2.7. Determination of Non-Specific Immune Parameters and Antioxidant Enzymes

The lysozyme activity (LZM, U mL^−1^) in the hemolymph was measured by detecting the decrease in turbidity after the lysis of the Gram-positive bacterium *Micrococcus lysodeikticus*, following the turbidimetric method of Ellis [35] and Van Doan et al. [36]. The activities of the antioxidant enzymes, including superoxide dismutase (SOD, U mL^−1^), were examined spectrophotometrically at 490 nm according to the method of Misra and Fridovich [37], and catalase (CAT, U mL^−1^) was examined spectrophotometrically at 450 nm following Luck [38] and Panase et al. [39]. Lipid peroxidation in terms of malondialdehyde (MDA, nmol mL^−1^) was measured by the thiobarbituric acid reactive substances (TBARS) method [40].

### 2.8. Investigation of Biofloc Organisms Using Metagenomic Analysis

A 400 mL biofloc water sample was filtered through 0.22 µm polyethersulfone filter membranes to obtain the microbial cells. The filtered membranes were then processed for metagenomic DNA extraction using a DNeasy PowerWater kit (QIAGEN GmbH, Hilden, Germany) according to the manufacturer’s protocol. The quantity and quality of the extracted DNA were measured using a NanoDrop™ One/OneC Microvolume UV-Vis Spectrophotometer (Thermo Fisher Scientific). The extracted DNA was then preserved at −80 °C.

For bacterial sequencing, the 16S rRNA (V3-V4 region) gene was amplified using polymerase chain reaction (PCR) with specific primers (341F: 5′CCTAYGGGRBGCASCAG 3′ and 806R: 5′GGACTACNNGGGTATCTAAT 3′). For eukaryotic sequencing, the 18S rRNA (V9 region) gene was amplified with specific primers (1380F: 5′CCCTGCCHTTTGTACACAC 3′ and 1510R: 5′CCTTCYGCAGGTTCACCTAC 3′). All the PCR reactions were carried out with 15 μL of Phusion^®^ High-Fidelity PCR Master Mix (New England Biolabs Inc., Ipswich, MA, USA), 2 μM of forward and reverse primers, and 10 ng of template DNA. Thermal cycling consisted of an initial denaturation at 98 °C for 1 min, followed by 30 cycles of denaturation at 98 °C for 10 s, annealing at 50 °C for 30 s, and elongation at 72 °C for 30 s. Finally, an elongation step at 72 °C for 5 min was carried out.

A volume of 1x loading buffer (containing SYB green) was mixed with the PCR products, and electrophoresis was performed on 2% agarose gel for detection. The PCR products were mixed in equidensity ratios, with the mixtures purified using a Qiagen Gel Extraction Kit (Qiagen, Germany).

Paired-end reads were assigned to samples based on their unique barcodes and truncated by cutting off the barcodes and primer sequences. The paired-end reads were merged using pandaseq (version 2.1.1, https://github.com/neufeld/pandaseq) [41]. The merged sequences were then clustered using CD-HIT and the chimeric sequences were removed using VSEARCH (version 2.15.0). The taxonomy assignment of bacterial OTUs was performed using QIIME2’s classify-sklearn v. 2020.2.0 with a confidence level of 0.7 against the 16S rDNA database Greengenes. For classifying fungal 18S sequences, QIIME2’s classify-sklearn was performed with a confidence of 0.7 against the PR2. Composition visualization was performed.

### 2.9. Statistical Analysis

Data were analyzed using one-way analysis of variance (ANOVA). Significant differences at *p* < 0.05 were detected by Duncan’s new multiple range test. Data variances were reported as the standard error (S.E.) of the mean of three replicates.

## 3. Results

### 3.1. Biological Characteristics

The biology of the fairy shrimp was investigated under various dietary conditions encompassing *Chlorella vulgaris*, biofloc, *Spirulina* sp., and food powder, yielding diverse outcomes. Mean values, standard errors, and ranges are presented in Table 1.

The lifespan of male fairy shrimp differed significantly among the dietary groups (*p* < 0.05). The longest lifespan was observed in the biofloc group (44.00 ± 6.00 days), significantly higher compared to the food powder group (29.66 ± 4.17 days). The *Chlorella vulgaris* group recorded 28.33 ± 1.20 days, while the *Spirulina* sp. group had the lowest value of 23.66 ± 3.38 days. For female fairy shrimp, the biofloc group had the longest lifespan 44.33 ± 4.67 days, followed by *Chlorella vulgaris* (31.00 ± 5.13 days) and *Spirulina* sp. (30.66 ± 9.70 days). The food powder group exhibited a significantly (*p* < 0.05) shorter lifespan of 18.00 ± 2.51 days.

The body length of male and female fairy shrimp was impacted by the dietary regimes. In males, the *Chlorella vulgaris* group had significantly (*p* < 0.05) longer body length at 28.06 ± 0.95 mm, with the biofloc group 25.66 ± 1.75 mm. The *Spirulina* sp. (18.70 ± 0.30 mm) and food powder groups showed lower values. In females, similar trends were observed, with the biofloc group having a body length of 27.60 ± 0.35 mm and the *Chlorella vulgaris* group 27.36 ± 0.93 mm. The maturation time and brood frequency were not significantly different among the four dietary groups (*p* > 0.05).

The number of broods per female varied significantly among the dietary groups (*p* < 0.05). The biofloc group had the highest number of broods, while the *Chlorella vulgaris* and *Spirulina* sp. groups had lower values, while the food powder group exhibited the lowest number of broods.

The number of eggs per brood significantly differed among the dietary groups (*p* < 0.05). The *Chlorella vulgaris* and the biofloc groups recorded the highest number of eggs per brood, while the *Spirulina* sp. and food powder groups displayed lower egg numbers.

The total number of eggs per female for the whole lifespan varied significantly among the dietary groups (*p* < 0.05). The biofloc group had the highest total number of eggs (5726.33 ± 1518.11), while the *Chlorella vulgaris*, *Spirulina* sp., and food powder groups exhibited lower egg numbers.

The survival rates of fairy shrimp fed different diets were significantly different (*p* < 0.05). The groups fed *Chlorella vulgaris* and biofloc showed similar survival rates of 83.58% and 79.25%, respectively. These values were significantly higher than those of the group fed *Spirulina* sp., which showed a survival rate of 70.67%. The lowest survival rate was observed in the group fed food powder, with a survival rate of 53.75%.

The dietary conditions significantly influenced the lifespan, body length, maturation time, and reproductive parameters of the fairy shrimp. These results contribute to a comprehensive understanding of the potential impacts of different diets on the life history traits of fairy shrimp.

**Figure 1:**
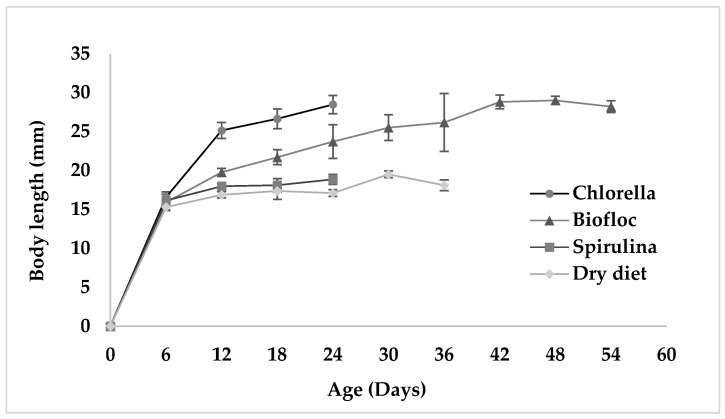
Growth of male fairy shrimps reared under different experimental diets.

**Figure 2:**
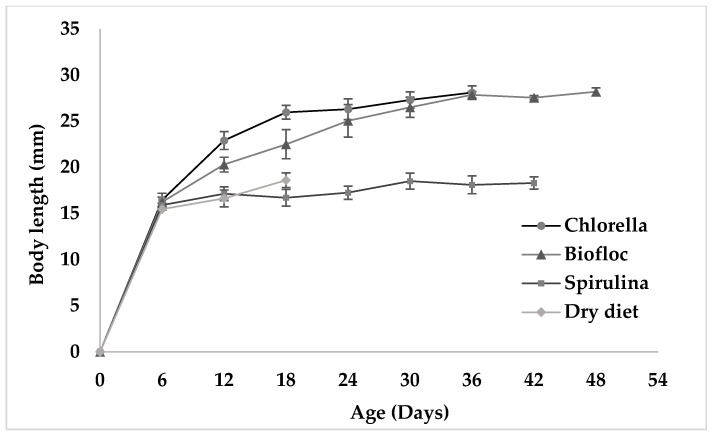
Growth of female fairy shrimps reared under different experimental diets.

The growth curves of fairy shrimp are represented in Figure 1 and Figure 2. The growth data of fairy shrimp indicated that both males and females grew more rapidly in *Chlorella vulgaris* and biofloc groups than the other experimental groups.

### 3.2. Proximate Composition and Carotenoid Content

The protein contents of the fairy shrimp were significantly different among the dietary groups (*p* < 0.05). The highest was recorded in the *Spirulina* sp. group (62.93 ± 0.45%), followed closely by the food powder group (61.89 ± 1%). The biofloc group (59.15 ± 0.59%) and the *Chlorella vulgaris* group (54.94 ± 1.72%) had lower protein contents (Table 2).

The lipid content was not significantly different among the dietary groups (*p* > 0.05), ranging from 4.45 ± 0.65% to 6.06 ± 0.75% across the *Chlorella vulgaris*, Biofloc, *Spirulina* sp., and food powder groups.

The ash content showed significant variations among the groups (*p* < 0.05). The highest ash content was recorded in the *Chlorella vulgaris* group (10.15 ± 0.79%), with *Spirulina* sp., food powder, and the biofloc groups displaying lower ash contents at 7.22 ± 0.22%, 8.07 ± 0.47%, and 8.23 ± 0.39%, respectively.

The carotenoid content exhibited statistically significant differences (*p* < 0.05). A high concentration of carotenoids was observed in the *Chlorella vulgaris* (273.27 ± 10.25 µg g^−1^), the *Spirulina* sp. (272.90 ± 8.10 µg g^−1^), and the biofloc (265.88 ± 11.38 µg g^−1^), while the food powder group had the lowest carotenoid content (86.72 ± 8.27 µg g^−1^) (Table 2).

### 3.3. Non-Specific Immune Parameters and Antioxidant Activities

The non-specific immune responses and antioxidant activities of fairy shrimp fed on different diets are shown in Table 3. Enzyme catalase activity was not significantly impacted by the different diets (*p* > 0.05). The lysozyme activity was significantly higher in fairy shrimp fed *Spirulina* sp., biofloc, and *Chlorella vulgaris* than those fed with food powder (*p* < 0.05). The superoxide dismutase (SOD) activity was significantly higher in fairy shrimp fed with *Chlorella vulgaris*, followed by biofloc, *Spirulina* sp., and the food powder, respectively (*p* < 0.05). The highest lipid peroxidation, expressed as MDA level, was observed in the food powder group.

### 3.4. Microorganism in Biofloc System

Gene sequencing of 4307 sequences was applied to evaluate the bacterial diversity and eukaryotic composition of biofloc. The total classified OTUs in the bacterial communities numbered 4152, while the eukaryotic community had 155 OTUs.

**Figure 3:**
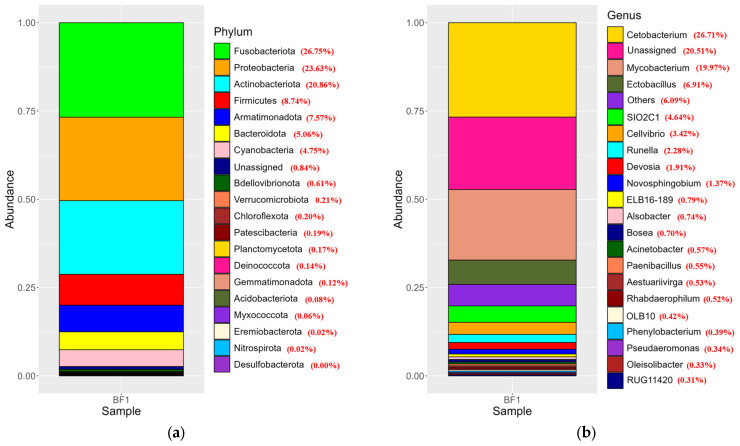
Taxonomic composition of microbial communities in the biofloc system based on 16S rRNA gene sequencing. (**a**) Distribution at the phylum level and (**b**) genus of organisms identified using the 16S rRNA gene.

The 16S rRNA gene analysis of the microbial taxa in biofloc sample showed that the highest relative abundance of bacterial organisms belonged to the phylum Fusobacteriota 26.75%, followed by phylum Proteobacteria 23.63%, and phylum Actinobacteriota 20.86%, with phyla Firmicutes and Armatimonadota contributing 8.74% and 7.57%, respectively to the overall microbial composition (Figure 3a). The top three bacterial genera found in the biofloc sample were *Cetobacterium* (26.71%), *Mycobacterium* (19.97%), and *Ectobacillus* (6.91%) as the main components (53.59%) (Figure 3b).

**Figure 4:**
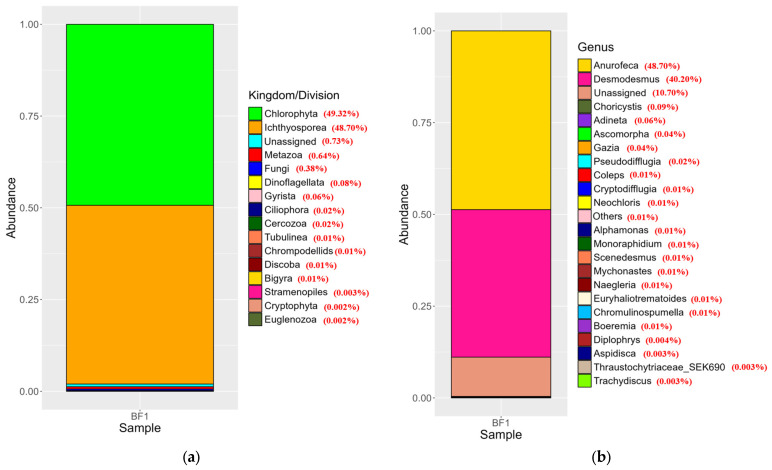
Taxonomic composition of organism in the biofloc system based on 18S rRNA gene sequencing. (**a**) Distribution at the phylum level and (**b**) genus of organisms identified using the 18S rRNA gene.

The analysis revealed varying relative abundance percentages for different eukaryotic taxa in the 18S amplicon. The eukaryotic organisms in the biofloc structure comprised algae, protozoa, metazoan, and fungi. The divisions Chlorophyta and Ichthyosporea were the predominant components, constituting 49.32% and 48.70%, respectively. Metazoa and fungi were found in smaller proportions, with relative abundances of 0.64%, and 0.38% (Figure 4a). At the genus level, the top three eukaryotic taxa were *Anurofeca* (48.70%), *Desmodesmus* (40.20%), and *Choricystis* (0.09%) (Figure 4b).

## 4. Discussion

This study investigated the biological performance of Thai fairy shrimp cultured on four diets as live *Chlorella vulgaris*, biofloc, dried powdered *Spirulina*, and commercial powdered feed. The results demonstrated that fairy shrimp fed with live feeds, *Chlorella*, and biofloc, exhibited significantly higher growth rates and longer lifespans compared with those fed powdered *Spirulina* and commercial powdered diets. Survival rates were higher in the groups receiving live feeds than in those receiving non-living feeds. These findings suggested that live feed plays a critical role in enhancing growth performance, longevity, and survival in fairy shrimp culture.

The results concurred with previous studies, which reported that live microalgae are highly suitable for fairy shrimp cultivation. Feeding fairy shrimp with *Schizochytrium* [42] and *Chlorococcum humicola* [31] significantly improved growth and survival. By contrast, Sriphuthorn [43] and Chaoruangrit et al. [31] reported that using powdered feed or powdered *Spirulina* alone resulted in reduced growth and shorter lifespan in fairy shrimp. These authors suggested that powdered diets were more suitable as supplementary feeds rather than primary nutritional sources. One possible explanation is that live feeds stimulate feeding activity, are easier to digest, and remain suspended in the water column longer than inert feeds [44]. Fairy shrimp are filter-feeding organisms [45], and the ability of feed particles to remain suspended in the water column facilitates continuous feeding and increases food availability compared with inert feeds that tend to settle at the bottom.

Traditionally, live *Chlorella vulgaris* have been widely used as primary feed for fairy shrimp culture due to their high nutritional value and ease of cultivation. However, the mass culture of microalgae still faces several limitations. Insufficient light conditions reduce algal productivity, and alternative live microbial feeds are required that can be cultured under diverse environmental conditions. Previous studies investigated microorganisms such as yeast [15], effective microorganisms (EM) [46], and the photosynthetic bacterium *Rhodopseudomonas faecalis* [47] as alternative feeds for fairy shrimp culture. Among these, bacterial-based feeds have shown considerable potential by enhancing growth performance and survival while simultaneously improving water quality in aquaculture systems.

Biofloc technology offers a promising approach. Bioflocs consist of complex microbial aggregates including bacteria, algae, protozoa, zooplankton, and aquatic fungi [48]. These microbial communities serve as an important protein source and contribute to the nutritional requirements of aquatic organisms. Bacteria within bioflocs can convert nitrogenous waste and organic matter into microbial biomass rich in proteins and lipids, which can subsequently be consumed by cultured organisms [49,50]. Microbial communities in biofloc systems have been reported to produce bioactive compounds that promote growth, enhance survival, and stimulate immune responses in aquatic animals [51,52]. However, in the present study, these mechanisms were not directly measured, and therefore the observed improvements in growth and survival should be interpreted as associations rather than direct causal effects.

Similar beneficial effects were reported for *Artemia* culture under limited algal conditions, with improvements in biomass production, growth rate, and survival observed when biofloc was used as a feed source [53,54,55]. These findings indicated that biofloc can function as an effective live feed source for zooplankton culture.

Beneficial bacteria in biofloc systems produce organic acids that inhibit pathogenic microorganisms, thereby enhancing host immunity and survival [56]. Biofloc microorganisms also contribute to improved water quality and intestinal morphology, which increases digestive efficiency and promotes growth [57]. In this study, the enhanced growth and survival of fairy shrimp fed biofloc may be associated with to the diverse microbial community present within the biofloc system. Nevertheless, as microbial-host interactions were not experimentally tested, this interpretation should be considered as a plausible explanation rather than direct evidence. Microbial analysis revealed that bacteria were the dominant group, with as many as 4152 OTUs, while other organisms such as microalgae, zooplankton, protozoa, and aquatic fungi contributed 155 OTUs. This diversity corresponds well with the natural feeding behavior of fairy shrimp, which typically consume a wide variety of microorganisms including algae, protozoa, rotifers, bacteria, and other small organisms [17,58].

Reproductive performance was also strongly influenced by dietary treatment. Fairy shrimp fed with *Chlorella* and biofloc exhibited significantly higher fecundity than those fed powdered *Spirulina* or powdered feed. The biofloc-fed group produced the highest reproductive output, reaching up to 25 broods and 5726 eggs, 10–20 times higher than observed in the powdered diet treatments. Previous studies reported that live feeds positively influence fecundity and hatching success in aquatic organisms [59]. Similarly, Jusoh et al. [60] reported that female aquatic animals cultured in biofloc systems produced more eggs per clutch and exhibited improved reproductive performance, while Ekasari et al. [61] reported that African catfish, *Clarias gariepinus* broodstock cultured in biofloc systems exhibited 26% higher fecundity compared with those cultured in conventional systems.

In this study, bacterial phyla such as Fusobacteriota, Proteobacteria, and Actinobacteriota were dominant within the biofloc microbial community. These bacterial groups are commonly recognized as probiotic candidates that have been reported to contribute to host health and reproductive performance [62,63,64]. In the present study, their presence in the biofloc system was observed, however their specific functional roles in enhancing reproduction were not directly evaluated. Probiotic bacteria have been reported to modulate intestinal microbiota, influence gene expression related to reproduction, and stimulate ovarian development, thereby enhancing fecundity and reproductive success. *Cetobacterium* (Fusobacteriota) has been associated with enhanced host resistance to pathogens, while members of Proteobacteria produce antimicrobial compounds that inhibit pathogenic fungi, bacteria, and viruses [65,66]. Actinobacteriota are also recognized as probiotic microorganisms that can produce enzymes, antimicrobial substances, vitamins, and immunomodulatory compounds, which support aquaculture productivity [67]. However, these mechanisms were not examined in this study and are presented here as supporting interpretations based on previous literature.

The biofloc system also contains diverse eukaryotic organisms identified through 18S rRNA amplicon analysis, including microalgae, zooplankton, protozoa, and fungi. These organisms are known to play important ecological roles within biofloc systems by regulating nitrogen levels and maintaining microbial balance in aquaculture environments [68,69]. In this study, their presence was identified through sequencing analysis, but their functional roles were not quantified. This diversity contributes to the nutritional value of biofloc because fairy shrimp are omnivorous filter feeders that can consume a wide range of microorganisms, including algae, protozoa, rotifers, and bacteria [17,70]. Nanoplankton also play an essential role in supporting the growth and maturation of fairy shrimp [71], and the presence of diverse eukaryotic microorganisms within the biofloc provides a balanced natural diet that supports growth and reproductive performance. In this study, green algae accounted for 49.32% of the eukaryotic community, explaining the relatively high carotenoid content in fairy shrimp fed with biofloc.

Carotenoids are another important component associated with the nutritional value of biofloc. These pigments, commonly found in crustaceans and microalgae, function as antioxidants and immune modulators [72]. In aquaculture, carotenoids are widely supplemented in feeds to improve pigmentation, health status, and growth performance [73]. In this study, fairy shrimp fed biofloc exhibited total carotenoid levels comparable to shrimp fed *Chlorella vulgaris* and *Spirulina* sp., both of which have high carotenoid content. This finding may be related to the high abundance of green algae within the biofloc community, accounting for nearly half of the detected eukaryotic organisms. Carotenoids are also precursors of vitamin A and play an essential role in immune function, growth, and larval development of aquatic organisms [74].

The immunological responses observed in this study further support the benefits of biofloc feeding. Lysozyme activity, an important indicator of innate immune response in aquatic animals, was significantly higher in fairy shrimp fed on *Chlorella vulgaris*, biofloc, and *Spirulina* diets compared with those fed powdered feed. Lysozyme functions as an antibacterial enzyme that can lyse bacterial cell walls, and plays a key role in the non-specific immune defense of aquatic organisms [51,75]. This enhanced immune may be associated with the presence of carotenoids and probiotic bacteria in biofloc, which both stimulate immune functions.

Fairy shrimp antioxidant responses were also impacted by the dietary treatments. Superoxide dismutase (SOD), a key antioxidant enzyme that protects organisms from oxidative stress [76], was significantly higher in fairy shrimp fed *Chlorella vulgaris*, biofloc, and *Spirulina* sp., while malondialdehyde (MDA), an indicator of lipid peroxidation and oxidative stress, was lower in these groups compared with the powdered feed treatment. These results indicated improved antioxidant defense mechanisms in the biofloc-fed group. While carotenoids and microbial components may contribute to this effect, their specific roles were not tested and should be considered as potential contributing factors. Previous studies also reported that probiotic bacteria and microbial biomass in biofloc systems enhanced antioxidant enzyme activities in aquatic organisms [77,78].

Based on the results of this study, *Chlorella vulgaris* and biofloc can be considered the most suitable diets for fairy shrimp culture. Both diets resulted in improved growth performance, survival rate, reproductive output, and immune responses compared with *Spirulina* and formulated feed. Biofloc, in particular, showed advantages in fecundity and sustainability, indicating its strong potential for application in aquaculture. However, the variability in biofloc composition should be carefully considered. Biofloc systems are complex and dynamic, stable biofloc quality can be maintained through proper management practices. These include maintaining an appropriate carbon-to-nitrogen (C:N) ratio (e.g., 15:1), continuous aeration, controlled feeding input, and regular monitoring of water quality parameters such as total ammonia nitrogen (TAN) and total suspended solids (TSS), as applied in this study.

Overall, the findings of this study are primarily based on observed biological responses. Mechanistic explanations regarding microbial interactions, probiotic effects, and reproductive enhancement are inferred from existing literature and should be interpreted with caution unless supported by further experimental evidence.

## 5. Conclusions

Feed type played a critical role in determining the growth performance, survival, reproductive capacity, and physiological responses of the Thai fairy shrimp. Among the four dietary treatments evaluated, biofloc and *Chlorella vulgaris* provided superior outcomes compared with *Spirulina* powder and commercial powdered feed. Fairy shrimp fed biofloc exhibited high growth performance and extended lifespan, with survival rates comparable to those fed *Chlorella vulgaris*, while showing significantly greater fecundity.

The results also indicated that fairy shrimp fed *Chlorella vulgaris*, biofloc, and *Spirulina* sp. possessed higher levels of total carotenoids, lysozyme activity, and antioxidant enzymes such as superoxide dismutase (SOD) and catalase (CAT), while exhibiting lower levels of oxidative stress, as indicated by malondialdehyde (MDA). By contrast, individuals fed powdered feed had lower immune and antioxidant enzyme activities and higher oxidative stress, indicating poorer physiological condition. These findings highlight the importance of nutritionally rich and biologically active feed sources in supporting the health and physiological stability of fairy shrimp.

The enhanced performance observed in the biofloc treatment was attributed to the diverse microbial and eukaryotic communities present within the biofloc matrix, including bacteria, microalgae, protozoa, and zooplankton. These organisms collectively provide a complex and nutritionally balanced food source that supports growth, reproduction, immune responses, and antioxidant capacity. Beneficial microbial communities in biofloc contribute to improved digestive efficiency, immune stimulation, and enhanced nutrient utilization. The biofloc used in this study originated from a tilapia biofloc culture system, with excess suspended solids periodically removed to maintain water quality. The reutilization of this biofloc biomass as feed for fairy shrimp represents a sustainable and cost-effective approach for aquaculture production. This strategy reduces organic waste while converting microbial biomass into a valuable feed resource.

Biofloc represents a promising alternative or supplementary feed for fairy shrimp culture rather than a complete replacement for conventional microalgal feeds such as *Chlorella vulgaris*. The application of biofloc enhanced growth, survival, reproductive performance, and physiological health in fairy shrimp, and also promoted sustainable aquaculture practices through resource recycling and waste reduction. Compared with *Chlorella*, biofloc provides a continuous and diverse natural food source while improving water quality through nutrient recycling. This makes it particularly suitable for large-scale aquaculture systems. However, optimization of biofloc composition and culture conditions is required to ensure consistent performance.

## Figures and Tables

**Table 1:** Biology of fairy shrimp reared with different diets, *Chlorella vulgaris*, biofloc, *Spirulina* sp. and food powder. Values followed by the same letter(s) are not significantly different (*p* > 0.05) (mean ± standard error and range).

Life History Characteristics	*Chlorella vulgaris*	Biofloc	*Spirulina* sp.	Food Powder	*p*-Value
Life span (days)					
Male	28.33 ± 1.20 ^b^ (26.00–30.00)	44.00 ± 6.00 ^a^ (38.00–56.00)	23.66 ± 3.38 ^b^ (17.00–28.00)	29.66 ± 4.17 ^b^ (25.00–38.00)	0.037
Female	31.00 ± 5.13 ^ab^ (24.00–41.00)	44.33 ± 4.67 ^a^ (35.00–49.00)	30.67 ± 4.18 ^ab^ (26.00–39.00)	18.00 ± 1.53 ^b^ (15.00–20.00)	0.014
Body length (mm)					
Male	28.06 ± 0.95 ^a^ (26.50–29.80)	25.66 ± 1.75 ^a^ (22.30–28.20)	18.70 ± 0.30 ^b^ (18.40–19.30)	17.43 ± 0.37 ^b^ (16.80–29.80)	0.000
Female	27.36 ± 0.93 ^a^ (25.90–29.10)	27.60 ± 0.35 ^a^ (26.90–28.00)	17.56 ± 0.35 ^b^ (16.90–18.10)	17.13 ± 0.93 ^b^ (16.20–19.00)	0.000
Maturation time (days)	8.33 ± 0.33 ^a^ (8.00–9.00)	8.33 ± 0.33 ^a^ (8.00–9.00)	8.33 ± 0.33 ^a^ (8.00–9.00)	9.00 ± 0.57 ^a^ (8.00–10.00)	0.596
Number of broods per female	12.00 ± 1.52 ^b^ (10.00–15.00)	25.00 ± 6.65 ^a^ (13.00–36.00)	10.00 ± 2.88 ^b^ (5.00–15.00)	7.00 ± 2.00 ^b^ (5.00–11.00)	0.045
Number of eggs per brood	186.02 ± 51.17 ^a^ (134.30–288.36)	229.57 ± 2.08 ^a^ (225.76–232.92)	39.64 ± 6.77 ^b^ (31.00–53.00)	32.57 ± 11.16 ^b^ (19.00–54.73)	0.001
Number of eggs per female	2182.00 ± 533.35 ^b^ (1343.00–3172.00)	5726.33 ± 1518.11 ^a^ (3028.00–8281.00)	366.33 ± 79.89 ^b^ (265.00–524.00)	272.33 ± 164.99 ^b^ (95.00–602.00)	0.005
Brood frequency (days)	1.88 ± 0.12 ^a^ (1.70–2.13)	1.40 ± 0.10 ^a^ (1.31–1.62)	1.93 ± 0.43 ^a^ (1.40–2.80)	1.59 ± 0.23 ^a^ (1.18–2.00)	0.480
Survival rate (%)	83.58 ± 2.34 ^a^ (80.00–88.00)	79.25 ± 2.50 ^a^ (74.50–83.00)	70.67 ± 1.22 ^b^ (68.25–72.25)	53.75 ± 2.50 ^c^ (50.00–58.50)	0.000

**Table 2:** Proximate composition and carotenoid content of fairy shrimp reared with different diets, *Chlorella vulgaris* biofloc *Spirulina* sp. and food powder, n = 3. Values followed by the same letter(s) are not significantly different (*p* > 0.05) (mean ± standard error and range).

Parameter	*Chlorella vulgaris*	Biofloc	*Spirulina* sp.	Food Powder	*p*-Value
Protein (%)	54.94 ± 1.72 ^c^ (52.76–58.36)	59.15 ± 0.59 ^b^ (58.49–60.34)	62.93 ± 0.45 ^a^ (62.39–63.85)	61.89 ± 1.00 ^ab^ (60.16–63.63)	0.003
Lipid (%)	4.45 ± 0.65 ^a^ (3.32–5.59)	4.92 ± 0.21 ^a^ (4.60–5.33)	4.92 ± 0.16 ^a^ (4.62–5.19)	6.06 ± 0.75 ^a^ (5.00–7.54)	0.236
Ash (%)	10.15 ± 0.79 ^a^ (8.73–11.49)	8.23 ± 0.39 ^b^ (7.78–9.02)	7.22 ± 0.22 ^b^ (6.90–7.67)	8.07 ± 0.47 ^b^ (7.14–8.70)	0.022
Carotenoid content (μg g^−1^)	273.27 ± 10.25 ^a^ (255.94–291.45)	265.88 ± 11.38 ^a^ (248.59–287.36)	272.90 ± 8.10 ^a^ (261.66–288.64)	86.72 ± 8.27 ^b^ (74.34–102.43)	0.000

**Table 3:** Immune parameters and antioxidant activities of fairy shrimp reared with different diets, *Chlorella vulgaris* biofloc *Spirulina* sp. and food powder, n = 3. Values followed by the same letter(s) are not significantly different (*p* > 0.05) (mean ± standard error and range).

Parameter	*Chlorella vulgaris*	Biofloc	*Spirulina* sp.	Food Powder	*p*-Value
Catalase (U mL^−1^)	2.91 ± 0.41 ^a^ (2.50–3.75)	2.91 ± 1.10 ^a^ (1.25–5.00)	1.25 ± 0.72 ^a^ (0.00–2.50)	0.94 ± 0.31 ^a^ (0.00–1.25)	0.179
Lysozyme (U mL^−1^)	17.33 ± 1.52 ^a^ (12.00–22.00)	17.66 ± 1.74 ^a^ (10.00–22.00)	19.00 ± 1.12 ^a^ (14.00–22.00)	11.00 ± 1.52 ^b^ (6.00–16.00)	0.005
Superoxide dismutase (SOD) (U mL^−1^)	28.12 ± 1.08 ^a^ (26.25–30.00)	22.50 ± 1.08 ^b^ (20.63–24.38)	19.37± 0.62 ^bc^ (18.75–20.63)	18.75 ± 1.08 ^c^ (16.88–20.63)	0.001
Malondialdehyde (MDA) (µmol L^−1^)	15.99 ± 0.18 ^b^ (15.41–16.29)	15.51 ± 0.06 ^b^ (15.41–15.71)	15.80 ± 0.22 ^b^ (15.12–16.29)	19.62 ± 0.71 ^a^ (18.35–21.88)	0.000

## Data Availability

Data are contained within the article.
